# Assessment of Oral Health Literacy and Influencing Factors: Retrospective Analysis of Patients Treated by Dental Students

**DOI:** 10.1111/eje.13122

**Published:** 2025-05-21

**Authors:** Alicia Maria Meyer‐Hofmann, Anna Greta Barbe, Max von Kohout, Ghazal Aarabi, Isabel Deeg, Michael Jochen Wicht, Sonja Henny Maria Derman

**Affiliations:** ^1^ Faculty of Medicine and University Hospital Cologne Polyclinic for Operative Dentistry and Periodontology, University of Cologne Cologne Germany; ^2^ Institute of Medical Statistics and Computational Biology University of Cologne, Faculty of Medicine and University Hospital Cologne Germany; ^3^ Department of Periodontics, Preventive and Restorative Dentistry, Center for Dental and Oral Medicine University Medical Center Hamburg‐Eppendorf Hamburg Germany

**Keywords:** communication skills, dental education, oral health behaviour, oral health literacy, periodontal therapy

## Abstract

**Introduction:**

Oral health literacy (OHL) plays a crucial role in determining oral health outcomes, particularly in preventing and managing oral health issues. It is primarily developed through communication between dentists and patients—a key focus in dental education. To enhance this training, understanding OHL in student teaching is essential. This study aimed to assess OHL among patients undergoing treatment performed by dental students and identify influencing factors.

**Materials and Methods:**

This retrospective analysis evaluated the Oral Health Literacy Profile (OHLP) of patients (≥ 18 years) treated by dental students in their fourth and fifth year. The OHLP measures oral health behaviour (OHB) and oral health knowledge (OHK) (scored 0–100). Patients were grouped by age and periodontal therapy status. Statistical analyses included ANOVA and linear regression modelling.

**Results:**

A total of 222 questionnaires (106 females; mean age 56.8 ± 16.4 years) were analysed. The mean OHLP was 54.4 ± 16.1. Significant differences (*p* < 0.05) in OHB and OHK were observed between age groups: the lowest OHB was in younger adults (47.9 ± 18.9), while the highest OHB (63.4 ± 22.9) and lowest OHK (29.1 ± 22.9) were in older seniors; OHB scores were higher in periodontal patients. The regression model identified age, female gender, periodontal therapy, and OHK as significant predictors of OHB (*R* ^2^ = 0.25, *p* < 0.001).

**Conclusion:**

Significant gaps in OHL were revealed, particularly among males, younger adults, older seniors, and those not undergoing regular periodontal therapy. Students should receive specialised training to recognise these varying levels of oral health knowledge, behaviour, and influencing factors, enabling them to effectively address these issues during counselling.

## 1 Introduction

The concept of oral health literacy (OHL) encompasses an individual's ability to obtain, process and understand oral health information to make well‐informed decisions, and the ability to apply this information to one's own personal circumstances [1]. Oral health literacy (OHL) is becoming increasingly important within the field of dentistry [2, 3], due to the link between low levels of OHL and poor oral health outcomes [4, 5]. Individuals with inadequate OHL are more likely to have poor oral health outcomes as a result of inappropriate oral health behaviours and dental care utilisation. As a consequence, they demonstrate poorer oral health behaviours, are at an increased risk of developing gingivitis and periodontitis, are more likely to cancel dental appointments, and exhibit a higher prevalence of dental caries, missing teeth, and edentulism [6, 7, 8, 9, 10]. These issues make the topic relevant for patients, but also for every practising dentist from a clinical and health economic point of view.

Several instruments are available to measure OHL, with most focusing on word recognition and comprehension. However, these tools often have a high administration burden, which presents a challenge in their practical application [11]. The Oral Health Literacy Profile (OHLP) [12] is a brief instrument that has been developed for the assessment of various aspects of OHL, particularly knowledge, self‐reported oral hygiene practices, and dental service utilisation. The tool is designed to identify individuals in practical settings who may benefit from oral health education [13]. Gaining comprehensive understanding of OHL in specific patient groups is crucial for enhancing oral health education programmes and tailoring them to individual needs within the framework of patient‐centered dentistry [14, 15]. A key research gap is the lack of clarity regarding the characterisation of patients treated in student courses in terms of OHL and influencing factors. Consequently, the optimal approach to training students to effectively address these aspects remains unclear.

Communication strategies and structured information transfer are an important part of changing health behaviour, for example with regard to nutrition or oral hygiene [16, 17]. In the context of patient‐centered care, the (graduating) dentist plays a pivotal role as a communicator, with a keen understanding of the importance of communication skills and a dedicated stance as a health advocate [18, 19, 20]. Dental graduates are expected to demonstrate the capacity to identify and categorise behaviours exhibited by patients that may be healthy or risky. They are also expected to demonstrate proficiency in supporting patients through the process of behavioural change, utilising a fundamental understanding of the available counselling and therapeutic options [19, 20]. To enable students to learn patient‐centered communication skills related to improving oral health status, it is essential for them to gain a comprehensive understanding of the specific and individual OHL needs of their patients during training—the patient population who will later be cared for by the graduating dentists.

Thus, the objective in this retrospective data analysis was to describe the OHL of patients undergoing dental treatment performed by fourth‐ and fifth‐year dental students, and to identify patient‐centered factors that influenced their OHL.

## 2 Materials and Methods

### 2.1 Ethics and Data Protection

This retrospective study was approved by the local ethics review board of the Medical Faculty of the University of Cologne. Data protection was guaranteed due to an anonymization process before statistical analysis was performed. The data that support the findings of this study are available from the corresponding author upon reasonable request.

### 2.2 Study Population

From 1st October 2023 to 1st February 2024 (corresponding to the winter semester 2023/24), all patients aged ≥ 18 years who were being treated by the fourth‐ and fifth‐year dental students were routinely invited to complete a questionnaire on their OHL. All students followed the longitudinal communication curriculum that had been implemented since their first year [21]. The questionnaire was completed by each patient in writing on a voluntary basis during their introductory visit to the clinical student course in Operative Dentistry and Periodontology. Only questionnaires in which > 70% of the questions were answered by the patient were included in the analysis. The interim pseudonymization process was implemented with the objective of identifying and deleting duplicated entries, thereby ensuring that each patient was represented only once in the database.

### 2.3 Assessment of Oral Health Literacy

**FIGURE 1:**
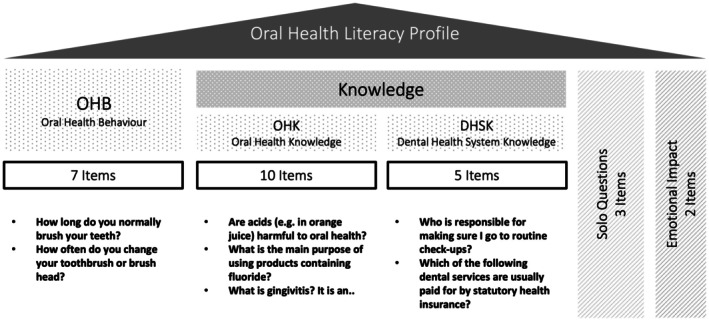
Overview of Oral Health Literacy Profile (OHLP) domains.

The short and validated instrument OHLP was used to assess each patient's OHL [12]. The OHLP is comprised of 28 items, with 22 core items divided into three categories: seven items that assess oral health behaviour (OHB), 10 items that assess oral health knowledge (OHK), and five items that assess Germany‐specific dental health system knowledge (DHSK). The three single questions refer to the reason for the responders' last dental visit, two self‐ratings of oral health knowledge and oral health status, and two questions that assess the emotional impact of costs and fear of pain. As treatment in the student course at the University of Cologne is provided only to German‐speaking patients or those accompanied by an accredited interpreter, the validated German version of the OHLP questionnaire was used in this study [12]. Figure 1 provides an overview of the OHLP structure. In the categories of OHB, OHK, and DHSK, a correct answer was assigned a value of one, while an incorrect answer or a non‐answer was assigned a value of zero. The total number of correct answers for each individual category was divided by the maximum number of possible points (seven for OHB, 10 for OHK, five for DHSK, and 22 in total) and multiplied by 100, resulting in a score (OHB, OHK, DHSK, total) on a scale of 0–100.

### 2.4 Patient Parameters

The following variables were extracted from the patient's file: age, gender, general illness, active pharmaceutical ingredients and the reason for the visit (check‐up, professional mechanical plaque reduction (PMPR), filling, root canal treatment, or active/supportive periodontal treatment) on the day the questionnaire was administered. In the clinical student course, a series of parameters were assessed on a routine basis during every appointment, including the number of teeth, the decayed, missing, and filled teeth (DMFT) index, and the periodontal screening index (PSI, german adaption of the CPITN).

### 2.5 Population Grouping

The population was categorised by age. Age grouping was made in accordance with those described in the DMS V [22]. The age groups in the DMS V [22] are based on the World Health Organisation (WHO) guidelines on oral health surveys [23] and include the age groups adults (aged 35–44 years), young seniors (65–74 years) and additionally older seniors (75–100 years). Age groups comprising individuals between the ages of 18–34 (young adults), 45–54, and 55–64 years (not previously defined in the DMS V [22] or by WHO [23]) were also established. Regardless of the treatment on the day the questionnaire was completed, patients were grouped into two categories: patients under active or supportive periodontal treatment (perio patients) and patients without periodontal treatment (other patients).

### 2.6 Statistical Analysis

Continuous variables are displayed as mean and standard deviation [mean (±SD)]. Categorical variables are displayed as absolute numbers and percentages [*n* (%)]. ANOVA was used to explore differences in OHB and OHK scores. A linear regression model was developed to predict OHB scores, with backward selection used to refine the model by removing non‐significant predictors. Significance levels were set at *p < 0.05, **p < 0.01, ***p < 0.001. Analyses were performed using IBM SPSS Statistics (V29) and R (version 4.3.2) for visualisation purposes.

## 3 Results

### 3.1 Included Questionnaires

**FIGURE 2:**
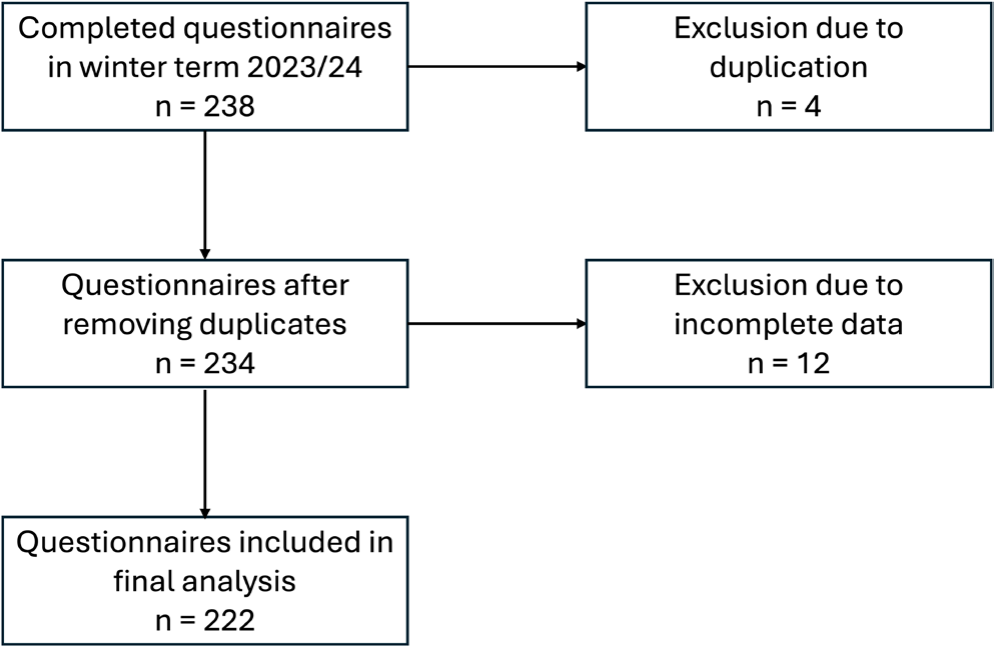
Flow chart of included questionnaires.

In total, 238 completed questionnaires were received at the end of the academic winter term (2023/2024). Four responses were excluded from the analysis because of duplication, and a further 12 were excluded due to incomplete data (≥ 30% of the questions were unanswered). Therefore, questionnaires from 222 patients (116 males, 106 females; mean age 56.8 ± 16.4 years) were included in the final analysis (Figure 2).

### 3.2 Sample Description

The most frequent reasons for the initial visit were preventive consultations with PMPR (*n* = 88, 40%) and periodontal appointments (*n* = 73, 33%). Patients had 24.4 ± 6.1 of their own teeth and a mean DMFT of 20.0 ± 5.5 (range 1–28). Of the 222 patients, 91 (41%) were classed as perio patients. Table 1 outlines the characteristics of the patients whose questionnaires were included.

**TABLE 1:** Patient and clinical dental parameters.

	Patients in the student course (*N* = 222)
Age (years), mean ± SD (range)	56.8 ± 16.4 (19–87)
Sex, *n* (%)	
Males	116 (52.3)
Females	106 (47.7)
Comorbidities, mean ± SD (range)	*n* = 220 1.5 ± 1.9 (0–14)
Active Pharmaceutical Ingredients, mean ± SD (range)	*n* = 221 2.0 ± 3.0 (0–18)
Reason for the visit, *n* (%)	
Check‐up	14 (6.3)
Professional mechanical plaque reduction	88 (39.6)
Filling	33 (14.9)
Root canal treatment	14 (6.3)
Perio Appointment	73 (32.9)
Subgrouping by periodontal therapy, *n* (%)	
Perio patients (PP)	91 (41)
Other patients (OP)	131 (59)
Number of teeth, mean ± SD (range)	24.4 ± 6.1, (0–32)
Decayed, missing, and filled teeth, mean ± SD (range)	20.0 ± 5.5 (1–28)
Max. Periodontal Screening Index, mean ± SD (range)	2.9 ± 0.9 (0–4)

Abbreviation: SD, standard deviation.

### 3.3 Oral Health Literacy

The mean OHLP total score for all patients (*N* = 222) was 54.4 ± 16.1. Table 2 provides an overview of the OHLP and subdomain scores subgrouped by age and periodontal therapy status. The OHK score was the OHLP subdomain with the lowest mean score (41.8 ± 22.7). Patients aged 45–54 years showed the highest OHB scores (66.9 ± 16.9). Younger seniors (65–74 years) maintained the highest OHLP and OHK scores. Perio patients had higher OHB scores (65.0 ± 18.2) than other patients (57.6 ± 19.6), but other patients demonstrated slightly higher OHK (43.2 ± 23.3) and DSHK (71.2 ± 19.6) scores.

**TABLE 2:** OHLP Scores in patients stratified by age and periodontal therapy status.

Patients in the student course (*N* = 222)									
		Subgrouping: age (years)						Periodontal therapy	
OHLP score	Total (*N* = 222)	18–34 (*n* = 34)	35–44 (*n* = 10)	45–54 (*n* = 35)	55–64 (*n* = 74)	65–74 (*n* = 37)	75–100 (*n* = 32)	OP (*n* = 131)	PP (*n* = 91)
Total	54.4 ± 16.1	48.1 ± 18.3	47.3 ± 16.5	56.2 ± 15.4	57.5 ± 15.8	58.1 ± 12.3	49.6 ± 15.9	54.1 ± 16.7	54.5 ± 15.1
OHB	60.8 ± 19.5	47.9 ± 18.9	54.29 ± 17.6	66.9 ± 16.9	61.6 ± 19.3	64.9 ± 14.9	63.4 ± 22.9	57.6 ± 19.6	65.0 ± 18.2
OHK	41.8 ± 22.7	39.7 ± 24.8	36.00 ± 19.0	41.1 ± 20.7	47.6 ± 22.6	45.1 ± 19.2	29.1 ± 22.9	43.2 ± 23.3	39.6 ± 21.8
DHSK	70.5 ± 21.6	65.3 ± 24.3	60.00 ± 31.3	71.4 ± 21.9	71.6 ± 20.7	74.6 ± 21.9	71.3 ± 15.2	71.2 ± 19.6	69.5 ± 24.3

*Note:* Presented as mean ± standard deviation.

Abbreviations: DHSK, dental health system knowledge; OHB, oral health behaviour; OHK, oral health knowledge; OHLP, oral health literacy profile; OP, other patient; PP, perio patient.

### 3.4 Subgrouping by Age

**FIGURE 3:**
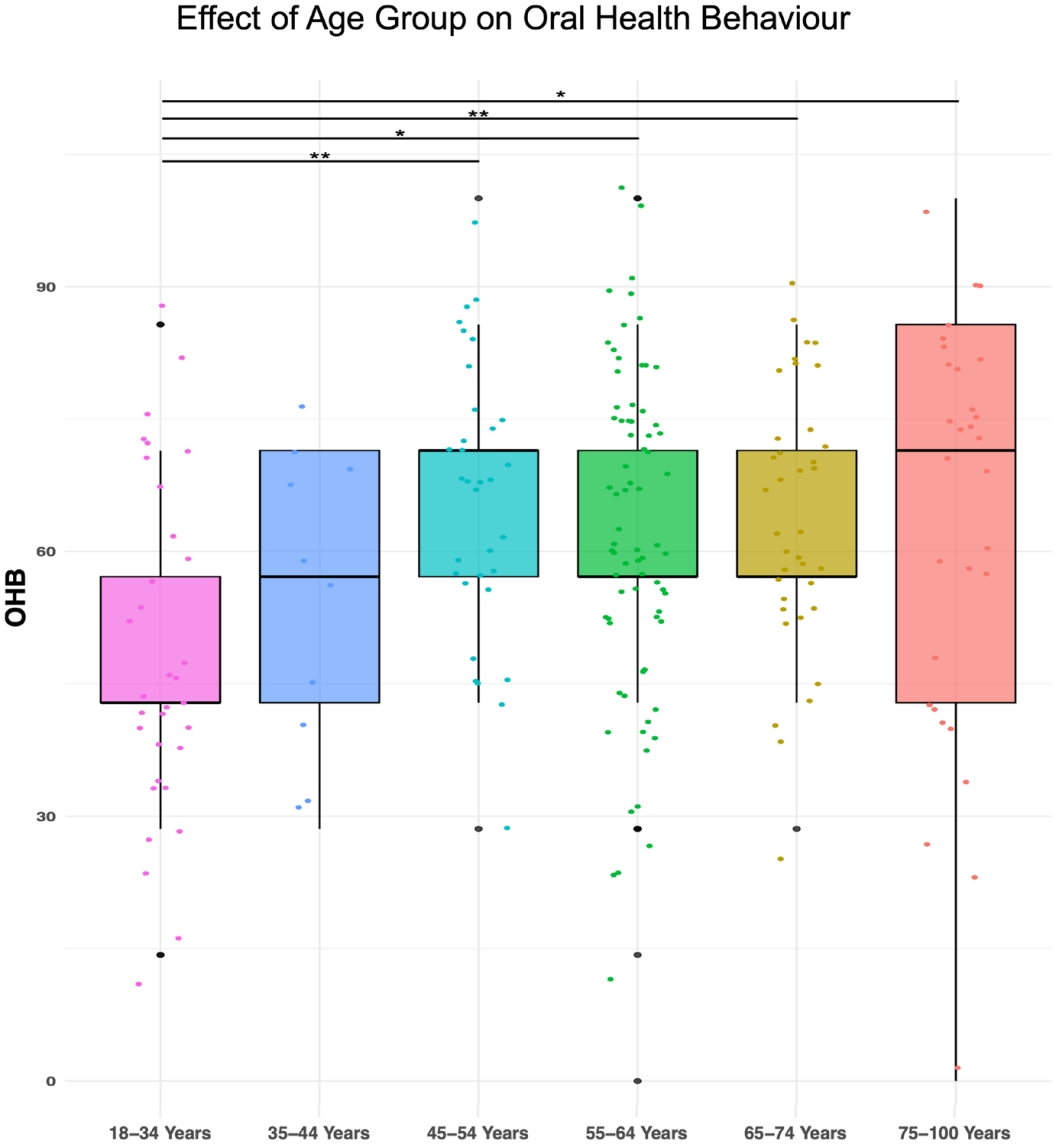
Group differences in oral health behaviour (OHB) by age. *p < 0.05, **p < 0.01.

The analyses of variance (ANOVA) revealed a significant effect of age group on the OHB score (*F*(5, 211) = 4.021, *p* < 0.001) (Figure 3). This indicates that there are statistically significant differences in the mean values of OHB across the six age groups, and a moderate proportion of variance attributed to the differences between age groups. The post hoc analysis revealed significant differences (*p* < 0.05) in OHB scores between the lowest OHB in the 18–34 age group and higher OHB in the following age groups: 45–54 years (17.3, 95% confidence interval (CI) 4.1, 30.6), 55–64 years (13.1, 95% CI 1.84, 24.4), 65–74 years (16.4, 95% CI 3.5, 29.3), and 75–100 years (14.4, 95% CI 0.8, 27.9). The corresponding *η* ^2^ showed a moderate effect of 0.08, according to Cohen's guidelines [24, 25].

**FIGURE 4:**
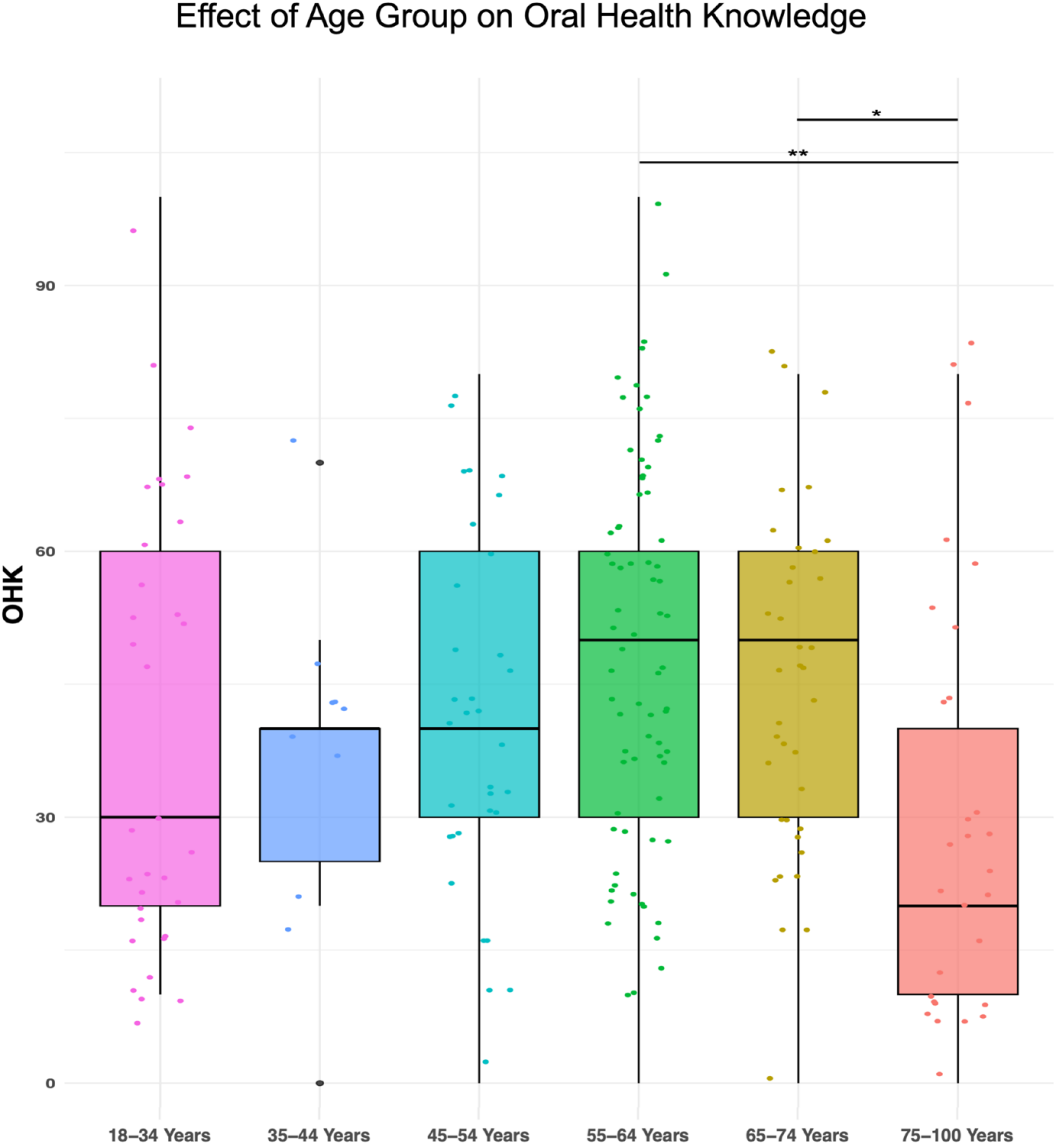
Group differences in oral health knowledge (OHK) by age. *p < 0.05, **p < 0.01.

The second one‐way ANOVA revealed a significant effect of age group on the OHK score (*F*(5, 211) = 3.274, *p* = 0.0072) that explained a moderate amount of total variance (Figure 4). This indicates that there were statistically significant differences in the mean values of the OHK across the six age groups. Post hoc ANOVA comparisons were adjusted for multiple testing using Tukey's method. The level of OHK differed statistically significantly between the 55 and 64 age group and lower OHK in the 75–100 age group (−18.2, 95% CI −32.1, −4.4) and 65–74 and lower OHK in the 75–100 age group (−15.8, 95% CI −32.5, −0.1), with a corresponding *η* ^2^ of 0.07, indicating a moderate effect size.

### 3.5 Subgrouping by Periodontal Therapy

**FIGURE 5:**
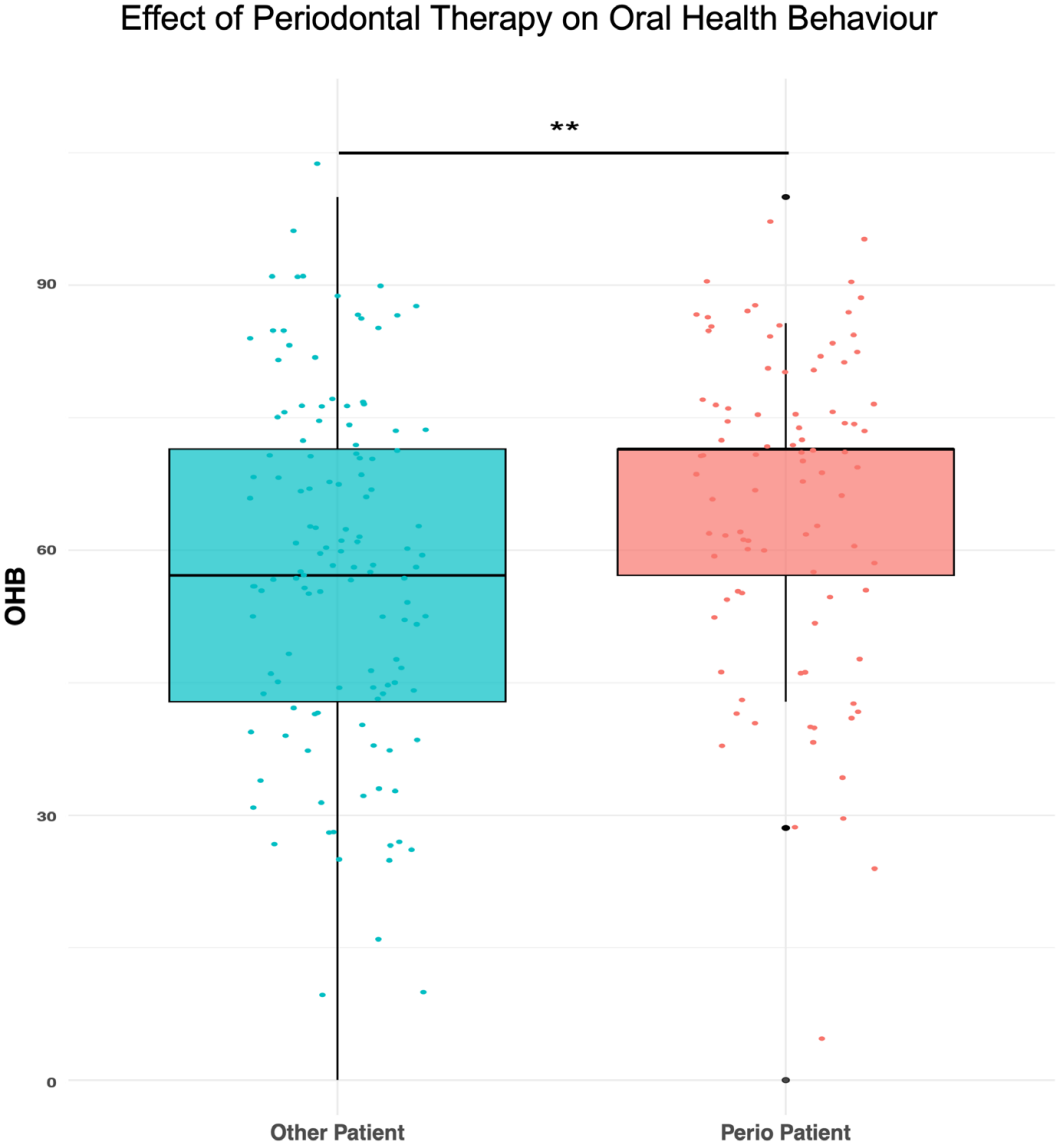
Group differences in oral health behaviour (OHB) by periodontal therapy. **p < 0.01.

For further analysis, a one‐way ANOVA was conducted to investigate whether there were any differences in OHB between the perio patient and other patient populations. The analysis indicated a significant effect of the perio patient subgroup on the outcome variable (*F*(1, 215) = 8.229, *p* = 0.00453) (Figure 5). Post hoc comparisons adjusted by the Tukey method showed a statistically significant difference in OHB levels between other patients and perio patients, with higher OHB in perio patients (difference 7.51 points, 95% CI 2.35, 12.7). This corresponds to an *η* ^2^ of 0.037, indicating a small effect size.

### 3.6 Predictors of Oral Health Behaviour

**FIGURE 6:**
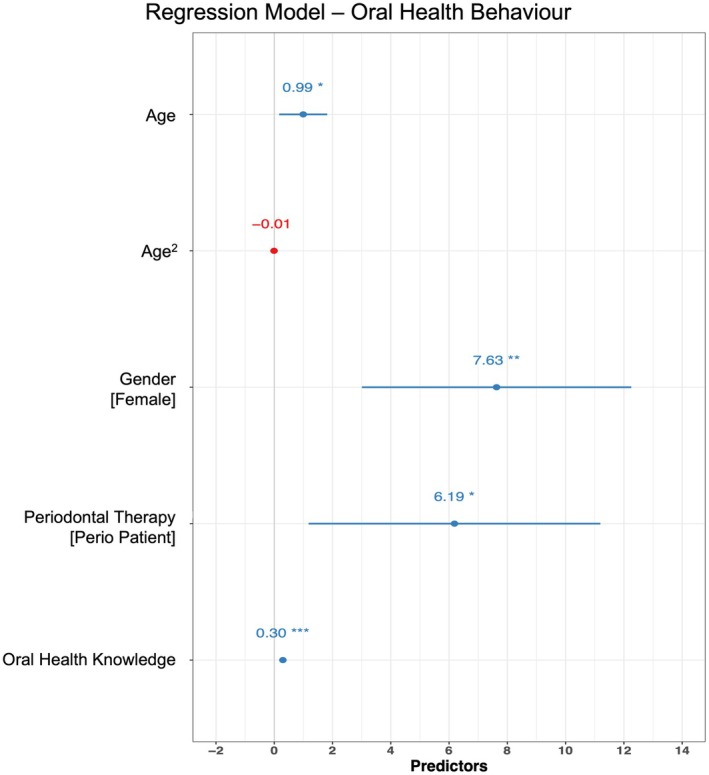
Results of a linear regression analysis examining the predictors of oral health behaviour (OHB). Coefficients were estimated using ordinary least squares regression, with 95% confidence intervals (CI) and *p*‐values calculated via a Wald t‐distribution approximation. *p < 0.05, **p < 0.01, ***p < 0.001.

The linear regression model indicated a statistically significant and moderate proportion of variance in OHB (*R* ^2^ = 0.25, *F*(5,211) = 14.02, *p* < 0.001, adjusted *R* ^2^ = 0.23), with an intercept of 10.66 (95% CI −10.49, 31.81, *t*(211) = 0.99, *p* = 0.321). Significant positive effects were found for increasing age (beta = 0.99, *p* = 0.019), female sex (beta = 7.63, *p* = 0.001), active and supportive periodontal therapy (perio patient) (beta = 6.19, *p* = 0.016), and higher OHK (beta = 0.30, *p* < 0.001). Age squared (Age^2^) had a non‐significant negative effect (beta = −0.007, *p* = 0.064). Detailed results are in Figure 6 and Appendix 1.

## 4 Discussion

We assessed the OHL of patients across all age groups, who had a broad range of reasons for attending the dental appointment with fourth‐ and fifth‐year dental students. This retrospective data analysis showed age‐related changes in OHB and in OHK. OHB was better in patients who received regular supportive or active periodontal therapy than in those who did not. The analysis revealed that males, patients not receiving regular active or supportive periodontal therapy, younger adults, and older seniors exhibited a need for improvement in their OHL.

The objective of the underlying study design—which employed a monocentric, retrospective data analysis approach—was to provide a description of the actual state of OHL measured by the OHLP in the patient population, rather than to assess changes in OHL through the implementation of an intervention. Across all three ANOVA models, similar challenges related to the underlying assumptions were encountered, primarily due to the complex measurement and the quasi‐grouping of the dependent variables. The subgroup analysis demonstrated the expected heterogeneity due to the differing conditions of treatment during the fourth and fifth years of the dental student course—patients require a greater investment of time, adhere to a schedule of appointments, and commonly make a lower financial contribution [26]. In comparison to the most extensive oral health study conducted in Germany, the patient population in this study exhibited slightly elevated DMFT values compared to the reference population [22, 27].

A linear regression model that explained 25% of the variance in OHB was developed. There were minimal differences between the *R* ^2^ (0.25) and the adjusted *R* ^2^ (0.23), indicating that the predictor selection was appropriate. Although some multicollinearity and non‐linearity were anticipated due to the quadratic age term, the model still demonstrated an ability to capture stagnation in OHB trends for older patients, despite a marginally non‐significant *p*‐value (0.06). However, homoscedasticity was not fully met for extreme values, likely due to measurement errors in the complex dependent variable (Appendix 2) and missing socioeconomic factors. Other studies have demonstrated the influence of income and educational level on OHL [28, 29]. Nevertheless, the model exhibited an adequate fit, supported by the parsimonious selection of meaningful predictors.

The mean OHLP values observed in this study were similar to the OHLP values observed in other studies investigating OHLP [12, 13]. In the current population, both knowledge scores were lower than those described by Spinler et al. [12] This could be due to differences in recruitment between the cohorts—the population in Spinler et al. [12] was recruited through online questionnaires, which causes a recruitment bias where individuals who have smartphones, use social media, or have technical knowledge and skills are more likely to access and complete the questionnaire, and may also have been able to take unlimited time to complete the questionnaire and use online searches to best complete the questionnaires. Patients in this study were presented with the questionnaire in its original paper format, without the option of preparation or the use of online searches.

Most studies report lower OHL in old age, as in this population [30, 31]. Parabolic relationships between OHL and age—with low OHL among younger adults and in old age, as seen in this study with low OHB in younger adults (18–34 years) and decreasing OHK in older seniors (75–100 years)—have not been described before. The lower level of OHL observed in our population of younger adults (aged 18–34) compared to age groups 45–54 years, 55–64 years, 65–74 years, and 75–100 years may be attributed to an increasing awareness of the necessity for optimal oral health practices with age. As dental problems increase with age—as illustrated by the DMFT index, for example—awareness of oral health problems increases. This motivates individuals to improve their oral health, which results in a change in behaviour [32]. Nevertheless, a significant reduction in OHL was observed among older seniors (aged 75–100), predominantly due to lower OHK. This prompts the question of whether the observed decline in OHK can be attributed to the fact that this generation was raised in an era when formal health education was not widely available. In the mid‐20th century, public health initiatives concentrated on infectious diseases to a greater extent than on chronic conditions or oral health, which resulted in older generations acquiring limited foundational health knowledge, including oral hygiene practices [33, 34]. An alternative hypothesis is that the observed decline in knowledge may be attributed to natural age‐related declines in executive function and episodic memory, or alternatively to the increased prevalence of neurodegenerative diseases [35].

Besides the previously mentioned factors of age and sociodemographic characteristics, sex also influences OHL levels [28]. A review of the existing literature indicates that women tend to demonstrate superior OHL [28, 36, 37]. Females in our study also tended to have better OHB, contributing to higher OHL than in men. This may be attributed to an increased propensity to engage in preventive oral health behaviours, such as regular dental visits and adherence to oral hygiene routines [38].

Domain Three of the Graduating European Dentist Curriculum by the Association for Dental Education in Europe (ADEE) underscores the integration of patient‐centered care into dental education [18, 39]. Therefore, graduates should recognise patients' needs, values, and preferences, communicate effectively, and promote shared decision‐making to enhance OHL and, consequently, improve oral health outcomes. Beyond providing an overview of patients' OHL in clinical student courses, the findings of this study offer valuable insights for practical applications in dental education. The focus should be on consistently addressing the individual knowledge and needs of patients, ensuring that students are trained not only to recognise patient‐specific behavioural risk factors but also to identify gaps in patients' knowledge. To achieve this, students need to develop the ability to assess both behavioural and educational deficiencies, as well as the influencing contextual factors, while subsequently communicating information in an accessible, low‐threshold, and patient‐friendly manner. A suitable teaching format is ‘situated learning’ in clinical student courses as method of teaching recommended by ADEE [40]. In addition to the standard integration of the OHLP into the medical history, having students directly evaluate the OHL questionnaire at the start of the treatment session would allow for an immediate, needs‐based response to oral health behaviours and knowledge gaps, ensuring care is tailored to each patient's individual needs.

Communication in dentistry and dental schools plays a crucial role in patient satisfaction, concordance, and promoting behavioural changes, which individually and together promote oral health outcomes [41, 42]. Prior to its incorporation into statutory health insurance—and even before the expansion of periodontal therapy services covered by the statutory health insurance, which is largely based on the European Federation of Periodontology guidelines—the significance of communication in dentistry was firmly established as a core element of active and supportive periodontitis therapy within the curriculum for dental students [20, 21, 43]. The emphasis on communication in dentistry, particularly in the context of periodontal therapy, may be a pivotal factor contributing to the noteworthy superior OHB observed among perio patients in our population.

Our study had limitations. The selected retrospective study design based on routine data does not permit conclusions to be drawn regarding the potential influence of socioeconomic factors, including income, marital status and level of education. Moreover, the applicability of these findings to the general population is constrained by the inherent bias associated with the monocentric study design of patients visiting the dental school and the voluntary nature of questionnaire completion. For instance, an age bias is evident, given that most patients who undergo student treatment have a considerable amount of time to spare. These individuals are predominantly pensioners or job seekers. Additionally, there is a bias in terms of wealth, with the majority of patients who are either financially constrained or reluctant to bear the costs associated with dental health issues seeking out the student course. In Western countries, contextual factors—including those of individuals attending dental treatment in student courses—vary significantly depending on the university's location, demographics, socioeconomic status, urban–rural distribution, and the ratio of publicly to privately insured individuals. However, within Germany, student course populations are generally considered relatively homogeneous. Further research is needed to explore effective strategies for improving oral health literacy across diverse population groups. Furthermore, data regarding the correlation between oral health, as delineated by plaque and gingival indices, and OHL is lacking. This is due to the fact that a retrospective study design including only routine data from patient records were available for evaluation of these indices, and plaque and gingival indices were not consistently collected for each patient.

## 5 Conclusion

Our study yielded valuable insights into the oral health literacy of patients undergoing dental treatment by fourth‐ and fifth‐year students at the dental school. Males, patients not receiving regular active or supportive periodontal therapy, younger adults, and older seniors exhibited a need for improvement in their OHL. Dental students need to be aware that these groups might need greater support and education about their oral health. There was a notable discrepancy in the levels of knowledge across age groups, which was inadequate in all age groups. It is unclear whether the objective for older adults should be to enhance knowledge transfer or whether alternative strategies are required to address the discrepancy in OHL, potentially through the incorporation of supportive environments. This study highlights the importance of incorporating OHL assessment into dental education to address patients' individual needs. Training should focus on recognising individual behavioural and knowledge gaps while improving students' ability to communicate effectively within a patient‐centered framework.

## Ethics Statement

Ethical approval was obtained from the local ethics review board of the Medical Faculty of the University of Cologne (24‐1057‐retro).

## Conflicts of Interest

The authors declare no conflicts of interest.

## Data Availability

The data that support the findings of this study are available from the corresponding author upon reasonable request.

## Appendix 1 Regression Analysis of Predictors of Oral Health Behaviour

**Table:**

Oral Health Behaviour Score			
Predictors	Estimates	Confidence interval	*p*
Age (years)	0.99*	0.17–1.82	**0.019***
Male	*Reference*		
Female	7.63**	3.01–12.25	**0.001****
I (age (years)^2^)	−0.01	−0.01 – 0.00	0.064
OHK Score	0.30***	0.19–0.40	**< 0.001*****
Normal	*Reference*		
Perio Patient	6.19*	1.18–11.19	**0.016**
Observations	217		
*R* ^2^/*R* ^2^ adjusted	0.249/0.232		

*Note: *p* < 0.05, ***p* < 0.01, ****p* < 0.001.

Abbreviation: OHK, oral health knowledge.

### Detailed Information About the Statistical Model

The model explains a statistically significant and moderate proportion of variance in the oral health behaviour (OHB) variable (*R* ^2^ = 0.25, *F*(5,211) = 14.02, *p* < 0.001, adjusted *R* ^2^ = 0.23). The model's intercept, corresponding to Age = 0, Sex = male, Periodontal therapy = other patient, and OHK = 0, was at 10.66 (95% confidence interval (CI)‐10.49, 31.81, *t*(211) = 0.99, *p* = 0.321). The coefficients in the regression model represent the change in the dependent variable (OHB) associated with a one‐unit increase in the corresponding independent variable, holding all other variables constant. Within this model, the effect of Age was statistically significant and positive (beta = 0.99, 95% CI 0.17, 1.82, *η* ^2^ = 0.0649, *t*(211) = 2.37, *p* = 0.019; Std. beta = 0.15, 95% CI 0.01, 0.28), the effect of Squared Age (Age^2^) was statistically non‐significant and negative (beta = −7.20e‐03, 95% CI −0.01, 4.27e‐04, *η* ^2^ = 0.0255, *t*(211) = −1.86, *p* = 0.064; Std. beta = −0.10, 95% CI [−0.20, 5.82e‐03]), the effect of Sex [female] was statistically significant and positive (beta = 7.63, 95% CI 3.01, 12.25, *η* ^2^ = 0.0324, *t*(211) = 3.25, *p* = 0.001; Std. beta = 0.39, 95% CI 0.16, 0.63), the effect of periodontal therapy (perio patient) was statistically significant and positive (beta = 6.19, 95% CI 1.18, 11.19, *η* ^2^ = 0.0123, *t*(211) = 2.44, *p* = 0.016; Std. beta = 0.32, 95% CI 0.06, 0.58), and the effect of oral health knowledge (OHK) was statistically significant and positive (beta = 0.30, 95% CI 0.19, 0.40, *η* ^2^ = 0.1143, *t*(211) = 5.67, *p* < 0.001; Std. beta = 0.35, 95% CI 0.23, 0.47). The model's coefficients were estimated using ordinary least squares regression. The 95% CI and *p*‐values were computed using a Wald t‐distribution approximation (Figure 6 in the main document).

## Appendix 2

Model Diagnostic Plots Assessing Model Fit, Assumptions, and Potential Issues Such as Collinearity, Normality, Linearity, and Influential Observations.
